# Does Clinical Management Improve Outcomes following Self-Harm? Results from the Multicentre Study of Self-Harm in England

**DOI:** 10.1371/journal.pone.0070434

**Published:** 2013-08-01

**Authors:** Nav Kapur, Sarah Steeg, Roger Webb, Matthew Haigh, Helen Bergen, Keith Hawton, Jennifer Ness, Keith Waters, Jayne Cooper

**Affiliations:** 1 Centre for Mental Health and Risk, Centre for Suicide Prevention, University of Manchester, Manchester, United Kingdom; 2 Centre for Suicide Research, The University of Oxford, Department of Psychiatry, Warneford Hospital, Oxford, United Kingdom; 3 Derbyshire Healthcare NHS Foundation Trust, Royal Derby Hospital, Derby, United Kingdom; Federal University of Rio de Janeiro, Brazil

## Abstract

**Background:**

Evidence to guide clinical management of self-harm is sparse, trials have recruited selected samples, and psychological treatments that are suggested in guidelines may not be available in routine practice.

**Aims:**

To examine how the management that patients receive in hospital relates to subsequent outcome.

**Methods:**

We identified episodes of self-harm presenting to three UK centres (Derby, Manchester, Oxford) over a 10 year period (2000 to 2009). We used established data collection systems to investigate the relationship between four aspects of management (psychosocial assessment, medical admission, psychiatric admission, referral for specialist mental health follow up) and repetition of self-harm within 12 months, adjusted for differences in baseline demographic and clinical characteristics.

**Results:**

35,938 individuals presented with self-harm during the study period. In two of the three centres, receiving a psychosocial assessment was associated with a 40% lower risk of repetition, Hazard Ratios (95% CIs): Centre A 0.99 (0.90–1.09); Centre B 0.59 (0.48–0.74); Centre C 0.59 (0.52–0.68). There was little indication that the apparent protective effects were mediated through referral and follow up arrangements. The association between psychosocial assessment and a reduced risk of repetition appeared to be least evident in those from the most deprived areas.

**Conclusion:**

These findings add to the growing body of evidence that thorough assessment is central to the management of self-harm, but further work is needed to elucidate the possible mechanisms and explore the effects in different clinical subgroups.

## Introduction

Self-harm is a major health problem internationally and a common cause of presentation to hospital [1]. Although a number of clinical guidelines have been published [2]–[4] the evidence-base to guide management is sparse. The most recent systematic review in the field suggested that psychological therapy may be of benefit in preventing repeat episodes of self-harm [3]. However studies to date have been underpowered. Levels of recruitment have been variable and research findings may not therefore be generalisable to the whole population of individuals who come to the attention of services following self-harm. In addition, the treatments which hold some promise - for example, cognitive behavioural therapy, problem solving therapy and dialectic behaviour therapy [5] - are not widely available to individuals in routine healthcare settings [3].

Although observational studies may be prone to bias and do not permit causal inferences to be drawn, they have the advantage that they are carried out in ‘real world’ settings and can allow investigation of outcomes in the majority of patients. Analysing data collected routinely by health services, a so called ‘outcomes research’ approach, may help to inform service provision for self-harm [6]. Much of the work to date has focused on the possible protective effect of psychosocial assessment [7]–[9], but there are also some findings suggesting that referral to specialist follow up may be beneficial [6]. Investigators have not, in general, considered other aspects of management such as admission to hospital. Neither have they examined potential mechanisms of action. Some authors have suggested that the psychosocial assessment is in itself therapeutic [10], [11], others that it has an effect through enabling treatment and follow up by specialist services [9]. The effect of management is likely to vary between settings [9] and could be modified by socioeconomic factors [12], but again this has not been investigated.

In this study we set out to examine the association between hospital management and outcome in a large cohort of self-harm patients presenting to three centres in England. Our specific objectives were to:

1. Investigate the association between four aspects of management (psychosocial assessment, medical admission, psychiatric admission and referral for specialist community mental health follow up) and repetition of self-harm, taking into account clinical and demographic factors.
2. Consider whether any observed effects were due to the specific aspect of management being considered, subsequent management, or associated elements of care.
3. Examine how outcome following different types of management varied according to socioeconomic context.

## Methods

### Ethics Statement

The self-harm monitoring system in Oxford was approved by South Central – Berkshire National Research Ethics Service and Derbyshire Research Ethics Committee approved the study in Derby. Both were granted ethical approval to collect data for both local and multicentre projects. In Manchester the project was reviewed by South Manchester Research Ethics Committee and was deemed not to require approval as the monitoring is conducted as part of a clinical audit system. All centres have approval under section 251 of the NHS Act (2006) to collect patient identifiable data without patient consent and to send patient details to the Data Linkage Service.

### Study Design and Setting

The study data were collected prospectively through the Multicentre Study of Self-Harm in England [13], a collaboration between three centres in Oxford, Manchester, and Derby. Each centre has an established monitoring system to collect data on episodes of self-harm presenting to emergency departments. Information was collected from assessments carried out by psychiatric and/or emergency department staff. Data included socio-demographic information, clinical factors such as previous psychiatric treatment and self-harm, details of the self-harm episode itself, and subsequent management. Standard definitions of self-harm were used across centres to include all acts of ’intentional self-poisoning or self-injury, irrespective of motivation’ [1].

Based on postcode of residence we also assigned an Index of Multiple Deprivation (IMD) score to each individual. The IMD is based on both national Census and administrative data sources and provides an overall measure of different aspects of area-level deprivation, including income, health and barriers to housing and services. Higher scores indicate greater levels of relative deprivation [14].

Service provision and catchment populations varied [13], but all centres had a seven day a week self-harm or mental health liaison team in place to provide specialist psychosocial assessments. The Manchester service was provided across three hospital sites. Out of hours cover in all centres was provided by junior psychiatrists or crisis teams. We were interested in four aspects of hospital management; psychosocial assessment by a mental health specialist, admission to a medical or psychiatric bed, specialist community mental health follow-up. Psychosocial assessment refers to an assessment of personal circumstances, social context, mental state, risk, and needs following self-harm [9]. Specialist mental health follow-up in this study included referrals to outpatient or community mental health teams and included crisis and drug and alcohol team referrals.

### Participants

Episodes of self-harm presenting to the participating emergency departments over a 10-year period from 1st January 2000 to 31st December 2009 (including those where the patient did not wait for assessment or treatment) were included. Episodes for patients aged under 16 years at the time of self-harm were not considered in the present study because the models of service provision for this group were distinct from those for adults.

### Outcomes

The main outcome for the current study was repeat self-harm within 12 months of an individual’s index episode during the study period. Repeat self-harm is a relatively common outcome [13], and is likely to indicate ongoing distress. It is also associated with an increased risk of suicide [15]. As such, repetition is regarded as one of the key outcomes for self-harm, and a 12-month time period has been used in a number of previous cohort and intervention studies [3], [16]. We did not ascertain repeat episodes in the community, neither did we identify repeat episodes presenting to hospitals outside the study centres. However, previous audits have indicated that episodes presenting to non-study hospitals would have a limited impact on the incidence of repetition. Repeat episodes of self-harm were identified by linking episodes to individuals through the centrally allocated National Health Service (NHS) number where available, or name and date of birth.

We did not consider suicide following self-harm as a specific outcome in relation to management for two reasons. First, such deaths can occur decades after a self-harm presentation [17] and the management an individual initially receives may not be strongly related to a poor outcome many years later. Second, since suicide is a rare outcome, the statistical power of these analyses would have been very low.

### Analysis

Service provision and catchment populations varied between centres, and so we analysed data for each centre separately. Because individual characteristics and initial method of self-harm were likely to be strong determinants of subsequent repetition risk [17] [18], all analyses were adjusted for differences in baseline demographic and clinical factors. Standard errors were corrected for clustering by hospital. Analyses were conducted using STATA V.11 and SPSS V.19. To investigate the impact of aspects of management on outcome we calculated hazard ratios for repetition within 12 months of the index episode for four aspects of management (specialist psychosocial assessment, medical admission, psychiatric admission, specialist mental health follow up) using Cox’s proportional hazards regression analysis.

We investigated potential mechanisms of the postulated effect of management on outcome by identifying aspects of management found to be associated with a lower risk of repetition in our initial analyses, and by adjusting the hazard ratios for other aspects of management. If the hazard ratios increased after adjustment then this might suggest that the variable adjusted for was partly responsible for the effect on outcome (that is, acted as a mediator). For example, if psychosocial assessment was found to be associated with a lower hazard ratio for repetition, but after adjustment for subsequent mental health follow up the hazard ratio increased towards unity, this could suggest that a possible effect of psychosocial assessment on repetition was mediated through its association with enhanced follow up. We also considered ‘clusters’ of management, in order to investigate whether these were associated with lower risk compared to single aspects of management. In addition, for any aspect of management found to be associated with lower risk of repetition, we considered repetition at 1, 3, and 6 months after the index episode. If an effect on repetition was causally related to the aspect of management itself, then we might expect the protective effect to attenuate over time.

To explore whether the effect of management varied by area-level socioeconomic context, we examined observed associations with aspects of management in low, medium and high deprivation groups. Low deprivation in this cohort spanned the 55.3% least deprived nationally and the high deprivation tertile was concentrated in the top 11.7% most deprived areas [14]. This indicated that our sample was somewhat more deprived than the general population of England.

## Results

### General Characteristics of the Sample

The sample consisted of 35,938 individuals presenting with 61,583 episodes of self-harm in the 10-year study period. Their median age was 30 years (IQR 21 to 40 years, range 16 to 97 years) and 20,527 (57.1%) were female. The most common method of harm at the index episode was self-poisoning with drugs (29,148 episodes, 81.1%) and the substances most commonly ingested in overdose (categories not mutually exclusive) were pure paracetamol (13,436 episodes, 46.1%), antidepressants (7,151 episodes, 24.5%), and benzodiazepines (3,613 episodes, 12.4%) (Table S1).

Overall 21,099 (58.7%) index episodes resulted in a psychosocial assessment, 1,861 (5.2%) in admission to a psychiatric bed, and 8,912 (24.8%) in a referral for specialist mental health follow up. Analyses of medical admission data were relatively complete in Centres B and C but restricted to a five-year period (2005 to 2009) in Centre A, because of data availability. In total, 14,935/24,405 (61.2%) of index episodes resulted in a medical admission.

With respect to repetition, 5,301 individuals (14.8%, 95%CI: 14.4% to 15.1%) repeated self-harm within 12 months of their index episode during the study period.

### Hospital Management and Risk of Repetition

Table 1 shows the association between management and risk of repetition within 12 months. We found that in two centres (B and C), receiving a psychosocial assessment was associated with a 40% lower risk of repetition relative to non-assessment. This was not the case in Centre A. In Centre C, but not Centre A and B, medical admission was associated with a slightly lower risk of repetition. Psychiatric admission or specialist mental health follow up was associated with a higher risk of repetition in most centres, and in three instances in Table 1 this elevated risk was statistically significant.

**Table 1:** Hospital management at index episode and relative risk of repetition within 12 months.

	Centre A		Centre B		Centre C	
	Number (%) repeating	Adjusted Hazard Ratio (HR)^1^ for repetition (95% CI)	Number (%) repeating	Adjusted HR^1^ for repetition (95% CI)	Number (%) repeating	Adjusted HR^1^ for repetition (95% CI)
**All**	2376/17 831 (13.3)		1372/8 402 (16.3)		1 553/9 705 (16.0)	
Specialist psychosocial assessment						
No	1265 (12.9)	1.0	333 (17.1)	1.0	497 (16.0)	1.0
Yes	1111 (13.9)	0.99 (0.90, 1.09)	1039 (16.1)	**0.59 (0.48, 0.74)**	1056 (16.0)	**0.59 (0.52, 0.68)**
General hospital admission 2						
No	486 (12.4)	1.0	190 (16.3)	1.0	854 (15.9)	1.0
Yes	575 (13.4)	**1.11 (1.02, 1.21)**	1182 (16.3)	1.04 (0.87, 1.24)	650 (15.9)	**0.81 (0.72, 0.90)**
Psychiatric admission						
No	2311 (13.2)	1.0	1199 (15.6)	1.0	1363 (15.3)	1.0
Yes	65 (18.4)	1.08 (0.81, 1.45)	173 (24.7)	**1.24 (1.05, 1.47)**	191 (23.8)	1.09 (0.93, 1.27)
Referred for specialist community mental health follow-up						
No	1863 (12.4)	1.0	748 (14.9)	1.0	979 (13.9)	1.0
Yes	513 (17.9)	**1.12 (1.01, 1.24)**	624 (18.4)	0.96 (0.85, 1.08)	575 (21.6)	**1.22 (1.09, 1.36)**

Statistically significant hazard ratios are highlighted in bold. These compare repetition in individuals receiving a particular aspect of management with repetition in all those not receiving that management (with the exception of ‘specialist community mental health follow-up’ where we exclude those with a psychiatric admission from the reference group).

1 Adjusted for baseline characteristics: main method of harm, drug/s used in self-poisoning (paracetamol/antidepressant/benzodiazepine), sex, age, ethnicity (White/Non-White/unknown), previous self-harm (yes/no/unknown), previous psych treatment (yes/no/unknown), current psych treatment (yes/no/unknown); standard errors and 95% CIs corrected for clustering by hospital.

2 The results for general hospital admission in Centre A are based on available data from a 5 year period, 2005 to 2009.

### Adjusting for other Aspects of Management

Table 2 shows the Hazard Ratios for psychosocial assessment by centre (adjusted for baseline characteristics) before and after further adjustment for other aspects of management. The hazard ratios showed little change after this further adjustment, so there was no indication that other aspects of management were mediating the relationship between psychosocial assessment and repetition found in Table 1.

**Table 2:** Psychosocial assessment and relative risk of repetition within 12 months adjusted for baseline characteristics plus other aspects of management.

	Hazard ratios (HRs) for repetition^1^			
	Psychosocial assessmentonly HR (95% CI)	Psychosocial assessment+medical admission HR (95% CI)	Psychosocial assessment+psychiatric admission HR (95% CI)	Psychosocial assessment+specialist community mental health follow-up HR (95% CI)
**Centre A**				
Not assessed	1.0	1.0	1.0	1.0
Assessed	0.99 (0.90, 1.09)	0.99 (0.92, 1.07)	0.98 (0.91, 1.07)	0.94 (0.88, 1.01)
**Centre B**				
Not assessed	**1.0**	**1.0**	1.0	1.0
Assessed	**0.59 (0.48, 0.74)**	**0.58 (0.46, 0.72)**	**0.60 (0.48, 0.75)**	**0.59 (0.47, 0.74)**
**Centre C**				
Not assessed	1.0	1.0	1.0	1.0
Assessed	**0.59 (0.52, 0.68)**	**0.63 (0.54, 0.72)**	**0.59 (0.52, 0.67)**	**0.56 (0.49, 0.64)**

Statistically significant hazard ratios are highlighted in bold.

1 Adjusted for baseline characteristics (please see Table 1 footnote).

In a separate analysis we considered three clusters of management: 1) psychosocial assessment and specialist community mental health follow up; 2) psychosocial assessment, medical admission, and specialist community mental health follow up; 3) psychosocial assessment and psychiatric admission. We found no indication that these clusters of management were associated with a lower risk of repetition than psychosocial assessment alone.

### Risk of Repetition by Time since Index Episode

Table 3 shows the relationship with psychosocial assessment over time. In only one of the centres (Centre B) did we find that psychosocial assessments were associated with a somewhat greater reduction in the risk of repetition in the short compared to the longer term. In this centre a psychosocial assessment was associated with a halving in the risk of repetition within one month of an index episode. The Schoenfeld test of proportional hazard showed that in this centre the association with psychosocial assessment varied significantly (p = 0.012) according to the time period for repetition under consideration.

**Table 3:** Psychosocial assessment and relative risk of repetition within different time periods.

	Hazard ratio (HR)^1^ for repetition within:		
	1 month	3 months	6 months
	HR (95% CI)	HR (95% CI)	HR (95% CI)
**Centre A**			
Not assessed	1.0	1.0	1.0
Assessed	1.09 (0.87, 1.37)	0.99 (0.87, 1.11)	1.03 (0.91, 1.17)
**Centre B**			
Not assessed	1.0	1.0	1.0
Assessed	**0.49 (0.34, 0.69)**	**0.57 (0.43, 0.75)**	**0.60 (0.47, 0.77)**
**Centre C**			
Not assessed	1.0	1.0	1.0
Assessed	**0.71 (0.56, 0.89)**	**0.64 (0.54, 0.77)**	**0.60 (0.51, 0.69)**

Statistically significant hazard ratios are highlighted in bold.

1 Adjusted for baseline characteristics (please see Table 1 footnote).

### Risk of Repetition by Level of Deprivation

**Figure 1:**
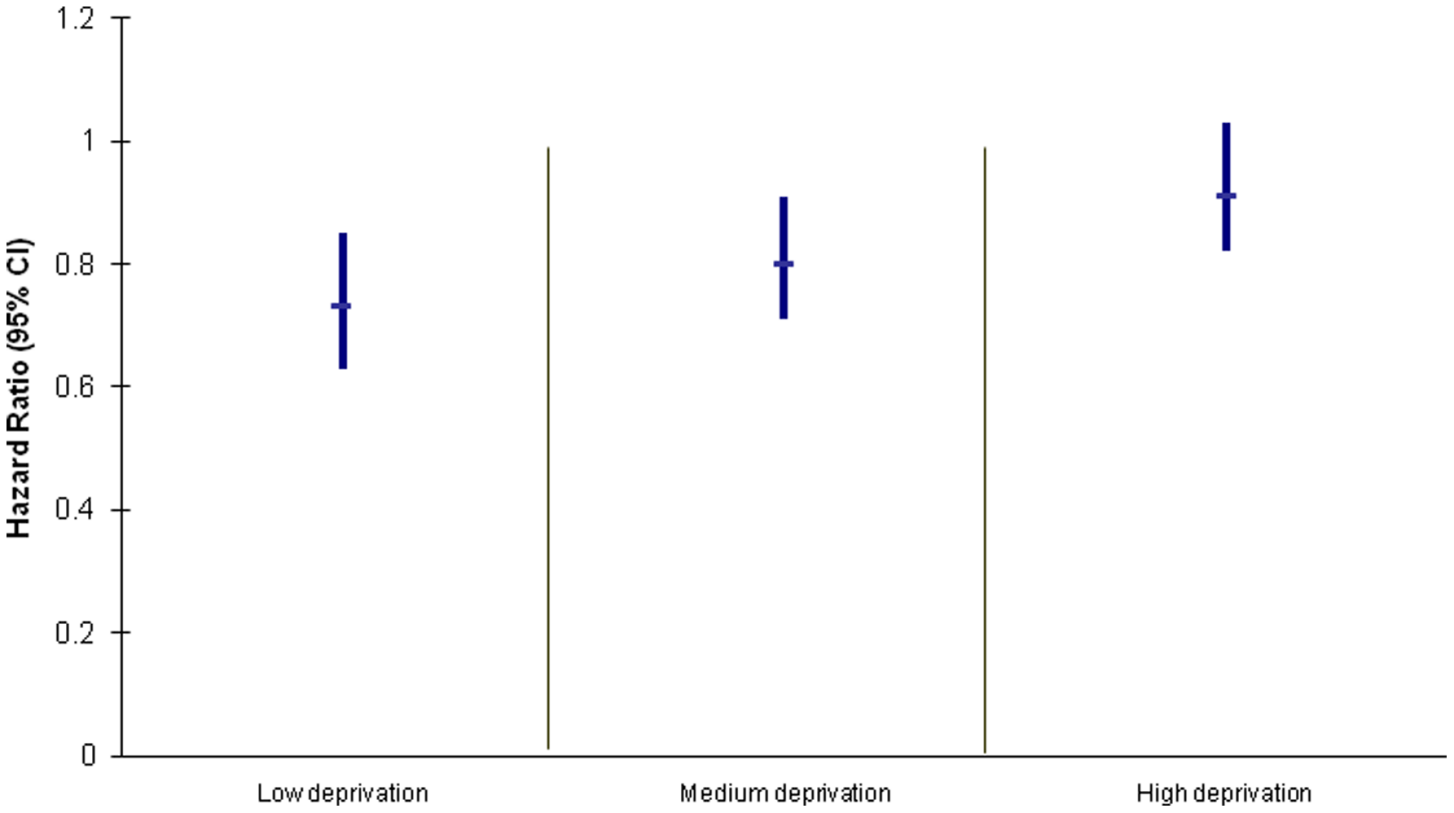
Psychosocial assessment and relative risk of repetition within 12 months by area level deprivation.

When we considered all centres together (Figure 1) there was visual evidence of a stepwise relationship between deprivation tertile of an individual’s area of residence and the effect of psychosocial assessment. Psychosocial assessment was associated with the smallest reduction in risk in individuals from the most deprived areas. Adjusting for centre made little difference to the results. We tested for evidence of a linear trend between deprivation score and hazard ratio for repetition in relation to psychosocial assessment by including deprivation score (as a continuous variable) as an interaction term. This did not quite reach the level of statistical significance (Wald chi-squared test = 3.53, p = 0.06).

## Discussion

### Main Findings

We found that one particular aspect of clinical management – provision of a psychosocial assessment by mental health staff – was associated with a 40% lower risk of repetition following self-harm in two of the three study centres after taking into account baseline demographic and clinical characteristics. Adjusting for other aspects of management made little difference to these results. We also found that several aspects of management considered together did not seem to exert a greater influence than assessment considered in isolation. In one centre, there was limited evidence that the possible short term effects of psychosocial assessment on repetition were greater than the long term effects. Our findings suggested that psychosocial assessment might have the least impact on those from the most deprived areas, but given the borderline significance of the linear trend, these data should be interpreted cautiously. Previous studies have examined the relationship between assessment and repetition, but the current study was one of the few to consider other aspects of management and the first to our knowledge to attempt to investigate possible mechanisms of action and the impact of deprivation.

### Methodological Issues

This is the largest study to date investigating the association between hospital management and outcome in a cohort of patients with suicidal behaviour. It involved collection of detailed individual-level clinical data, however, our findings should be interpreted in the context of some methodological limitations.

This was an observational study and although we found a number of important associations we are unable to make causal inferences. We cannot state with any certainty that it was the nature of care itself that accounted for the findings. Individuals were not allocated to differing management schemes randomly, but (presumably) on the basis of their underlying characteristics and clinical need. We tried to minimise the effects of this by adjusting for differences in important baseline clinical characteristics between the groups, but there may have been residual confounding factors that we did not measure. In this study we focused on producing readily interpretable estimates of treatment effects and, for simplicity, used standard risk-adjustment methods to address the issue of selection bias. However, we acknowledge that there are other analytical methods available that may further reduce bias in the estimates obtained from these data. These include use of the propensity score - to produce stratified estimates or to select participants for inclusion in the analysis – and instrumental variable analysis [19] [20].

Some patients may have repeated self-harm and attended an emergency department outside of the study area. An local audit of patient ‘cross-flows’ found that only a small minority of residents visited hospitals outside of the three participating Manchester sites. We estimated that we captured over 90% of self-harm presentations. Catchment areas in Oxford and Derby are more circumscribed than in Manchester [21], suggesting the overall impact in this study was likely to have been minimal. However, we acknowledge that hospital attendances outside of the study areas may introduce bias to our estimates.

Another potential weakness is that we considered only a limited number of aspects of immediate hospital management and did not collect data on non-hospital services or therapies offered after discharge from hospital. In addition, our mental health follow-up variable simply described referral for such follow up and we were unable to record whether patients actually attended.

We examined variation in associations between assessment and repetition by socio-economic status using a compositional area-based deprivation measure [14]. Of course, area-level deprivation may not necessarily correspond directly to an individual’s deprivation status. However, previous studies have suggested that people who self-harm have markers of individual-level deprivation that broadly reflect the areas where they live [22].

The study was carried out in three centres in England which were broadly representative of the styles of service provision in the UK [1], but the findings may not be generalisable elsewhere.

### Interpretation of Findings

If, as we and others have reported, psychosocial assessment after self-harm is associated with a reduced risk of repetition [7]–[9], then what might be the mechanism of this effect? We found little evidence in this study that it was linked to subsequent management or follow up arrangements, but our data were rather limited in this respect. In addition, psychological therapies shown to be associated with a reduced risk of repetition after self-harm are not widely available in most services [3]. Another possibility is that the assessment itself is therapeutic. Qualitative work suggests that one helpful aspect of assessment is the opportunity to talk through problems [10]. Assessment is experienced as most positive by service users when it involves good quality, hopeful engagement [10], [11], but also when offers of help from services translate into tangible actions [10]. A small randomised trial of young people following self-harm recently reported that levels of engagement with subsequent follow-up may improve after an enhanced ‘therapeutic assessment’ [23]. If a reduced risk of repetition in our study was related to the assessment itself, we might expect the effect to be strongest in the short term. There was a suggestion that this might have been the case in one of the three centres. Alternatively, could it be that the observed associations were accounted for simply by a group of high risk individuals who chose not to wait for assessment? This seems unlikely because the apparent protective effect of assessment in the two centres persisted even after individuals who did not wait were excluded (Centre B: HR 0.72, 95% CI 0.57 to 0.91. Centre C: 0.61, CI 0.53 to 0.70).

The possible beneficial effect of assessment was not seen in Centre A. Why might this have been the case? The proportion of individuals receiving a specialist psychosocial assessment varied between centres (Centre A 45%, Centre B, 77%, Centre C 68%), and our finding may reflect a selection effect based on a ‘high risk’ approach to management [9]. That is, only the patients at highest risk of future suicidal behaviour receive a specialist assessment in centres where the overall rate of assessment is low. Of course, this is contrary to national guidance which suggests that good quality assessment should be provided to all patients [3]. The professional background of the assessing clinician did not appear to explain the difference between centres - in all three centres, the majority of assessments were carried out by mental health nurses.

Some aspects of management (for example, medical or psychiatric admission) appeared to be associated with a greater likelihood of repetition in some centres, even after adjustment for baseline factors. We do not think this indicates that these aspects of care are harmful, but rather that it again may reflect a selection effect, whereby the highest risk individuals are given the most intensive forms of management.

The association between assessment and repetition appeared to vary by levels of socio-economic deprivation, although the linear trend across deprivation scores was not statistically significant. The impact of assessment may have been least in the most deprived areas. This could simply have reflected between-centre differences - Centre A (in which assessment was not associated with reduced repetition) contributed most individuals to the high deprivation group. However, it might also reflect the fact that individuals living in areas of high socioeconomic deprivation experience a variety of additional psychosocial stressors. The lack of effect of assessment in areas of high deprivation could also be a result of reduced help seeking or access to services. In this study, in all centres, those individuals who had self-harmed in the most deprived areas were the least likely to be in current psychiatric treatment.

### Research and Clinical Implications

It is unlikely that hospital management for self-harm has the same effect across the whole population of individuals who have self-harmed. Future studies need to examine the effect of aspects of management on important sub-groups (for example those in different age and ethnic groups, those with and without a past history of self-harm, those who self-injure compared to those who self-poison). Mortality (particularly suicide) should also be examined as an outcome, but of course such studies will have limited statistical power. Another focus of future work could involve exploring in more detail the appropriate ways to address selection bias when estimating treatment effects from these observational data, and might include the application of propensity score methods and instrumental variable analysis [19], [20]. Further work, perhaps using qualitative paradigms, is needed in order to add to our understanding of the possible mechanisms by which assessment might exert its effect [10] and identify the active ingredients of psychosocial assessment.

From a clinical perspective, our findings appear to highlight once again the central role of good quality assessment in the management of self-harm. They suggest that assessment by individual clinicians may make a tangible difference to outcome. This is a cause for therapeutic optimism in a group of patients who are often perceived as difficult to help by clinical services [24].

## Supporting Information

Table S1 **Baseline demographic and clinical characteristics of presenting individuals.** ^1^Categories not mutually exclusive. Most common medicine categories shown. ^2^Results for general hospital admission in Centre A are based on available data from a 5 year period, 2005 to 2009.
(DOCX)
