# Supplementary material for: Does Clinical Management Improve Outcomes following Self-Harm? Results from the Multicentre Study of Self-Harm in England
Source: PLoS One. 2013 Aug 1;8(8):e70434. doi: 10.1371/journal.pone.0070434 (PMC3731259; doi:10.1371/journal.pone.0070434)
Supplement: Table S1 — Baseline demographic and clinical characteristics of presenting individuals. 1Categories not mutually exclusive. Most common medicine categories shown. 2Results for general hospital admission in Centre A are based on available data from a 5 year period, 2005 to 2009. (DOCX) [file pone.0070434.s001.docx]

| **Characteristics of presenting individuals (no. of valid responses)** | **Number in cohort (%)** |
| --- | --- |
| Total individuals | 35,938 |
| ***Gender*** *(35,938)* |  |
| Male | 15,411 (42.9) |
| Female | 20,527 (57.1) |
| ***Age group*** *(35,938)* |  |
| Aged under 35 | 22,142 (61.6) |
| Aged 35+ | 13,796 (38.4) |
| ***Ethnic group*** *(35,938)* |  |
| White | 23,465 (65.3) |
| Non-White | 2,711 (7.5) |
| Unknown ethnicity | 9,762 (27.2) |
| ***Main method of harm*** *(35,938)* |  |
| Self-poisoning | 29,148 (81.1) |
| Self-cutting | 5,405 (15.0) |
| Other self-injury | 1,385 (3.9) |
| ***Medicines used in self-poisoning****^1^ (29,148)* |  |
| Paracetamol | 13,436 (46.1) |
| Benzodiazepine | 3,613 (12.4) |
| Antidepressant | 7,151 (24.5) |
| ***History of previous self-harm*** *(25,201)* |  |
| Any | 13,026 (51.7) |
| None | 12,175 (48.3) |
| ***Current treatment for psychiatric disorder*** *(26,502)* |  |
| Any | 9,932 (37.5) |
| None | 16,570 (62.5) |
| ***Previous treatment for psychiatric disorder*** *(25,743)* |  |
| Any | 12,128 (47.1) |
| None | 13,615 (52.9) |
| ***Management of individuals presenting to hospital*** |  |
| Specialist psychosocial assessment (35,938) | 21,099 (58.7) |
| General hospital admission ^2^ (24,405) | 14,935 (61.2) |
| Psychiatric admission (35,938) | 1,861 (5.2) |
| Referred for specialist community mental health follow-up (35,938) | 8,912 (24.8) |
